# Hyperedge bundling: Data, source code, and precautions to modeling-accuracy bias to synchrony estimates

**DOI:** 10.1016/j.dib.2018.03.017

**Published:** 2018-03-09

**Authors:** Sheng H. Wang, Muriel Lobier, Felix Siebenhühner, Tuomas Puoliväli, Satu Palva, J. Matias Palva

**Affiliations:** a Neuroscience Center, HiLife, University of Helsinki, Finland; b Doctoral Programme Brain & Mind, University of Helsinki, Finland; c BioMag laboratory, HUS Medical Imaging Center, Helsinki, Finland

## Abstract

It has not been well documented that MEG/EEG functional connectivity graphs estimated with zero-lag-free interaction metrics are severely confounded by a multitude of spurious interactions (SI), i.e., the false-positive “ghosts” of true interactions [1], [2]. These SI are caused by the multivariate linear mixing between sources, and thus they pose a severe challenge to the validity of connectivity analysis. Due to the complex nature of signal mixing and the SI problem, there is a need to intuitively demonstrate how the SI are discovered and how they can be attenuated using a novel approach that we termed hyperedge bundling. Here we provide a dataset with software with which the readers can perform simulations in order to better understand the theory and the solution to SI. We include the supplementary material of [1] that is not directly relevant to the hyperedge bundling per se but reflects important properties of the MEG source model and the functional connectivity graphs. For example, the gyri of dorsal-lateral cortices are the most accurately modeled areas; the sulci of inferior temporal, frontal and the insula have the least modeling accuracy. Importantly, we found the interaction estimates are heavily biased by the modeling accuracy between regions, which means the estimates cannot be straightforwardly interpreted as the coupling between brain regions. This raise a red flag that the conventional method of thresholding graphs by estimate values is rather suboptimal: because the measured topology of the graph reflects the geometric property of source-model instead of the cortical interactions under investigation.

**Specifications table**

**Table:** 

Subject area	*Physiology*
More specific subjects	*Neuroscience*
Type of data	*Human neuroimaging data*
How data was acquired	*Magnetoencephalography, MRI, see Honkanen et al., 2015*
Data format	*MEG forward-, inverse-operators, simulated functional connectivity graphs*
Experimental features	*Source time-series were generated to reflect the functional connectivity of simulated ground-truth graphs. These ground-truth time-series were next forward- and inverse-modeled to represent a virtual MEG measurement thus introducing linear mixing between sources. Next, functional connectivity was estimated from these source-modeled time-series using a zero-lag-free interaction metric. Finally, hyperedge bundling were performed and hyperedge sensitivity, specificity and separatablity were compared against that of raw metric edges.*
Data source location	*Helsinki, Finland*
Data accessibility	*The sample dataset, software executable and source code is publically available from below link to repository:*
	https://figshare.com/projects/Hyperedge_Bundling/26503
	*These are original DATA that have not been published elsewhere*

**Value of data**

• Allows the readers to replicate how one true interaction is “ghosted” into dozens of false-positive spurious interactions.
• Provides a platform to intuitively demonstrate how bundling of observed (spurious & true) edges into hyperedges decreases the false positive rate.
• Illustrates how mixing properties of a MEG/EEG source space are computed using simulations.
• Exposes an important fact that the source modeling accuracy biases functional connectivity estimates.

## 1 Data

The dataset includes the MEG forward, inverse and sparse-source-to-parcel-collapsing operators for 12 subject that were experimentally derived from the visual working memory experiment described below. We also provide 10^5^ randomly generated ground-truth graph templates for simulation purpose. Other data and group level graphs (described in the methods) can be created from aforementioned data with the software. The software can perform hyperedge bundling using connectivity graphs generated in this pipeline or using external data imported with compatible format (*.csv files).

## 2 Experimental design, material and methods

### 2.1 Toy model: A truncated Gaussian mixing function

For the toy model, we used a mixing function to simulate the mixing between sources. This mixing function assesses cross-talk by describing how activity at one source location is affected by leakage from other sources. Here, we used a 2D grid system of *n*×*n* point-sources to demonstrate how spurious interactions (SIs) arise when signals from interacting sources mix with their surrounding uncorrelated sources. The mixing function *f*_*mix*_(*v*_*i*_*,v*_*j*_) between sources *v*_*i*_ and *v*_*j*_ decreases with increasing spatial distance *d*_*ij*_ between them, so that:

(S1) $$f_{mix}\left(v_{i},v_{j}\right)=f_{0,dg}^{2}(d_{ij}),\;\mathrm{when}\;d_{ij}\leq 3d_{g},\;\mathrm{and}\;f_{mix}\left(v_{i},v_{j}\right)=0,\;\mathrm{when}\;d_{ij}>3d_{g},$$

where *f*_*0,dg*_^*2*^(*d*_*ij*_) is a truncated normal distribution (see Fig. 1A of [1]) with *μ*=0 and σ=*d*_*g*_ normalized so that the maximum mixing is a point-source's mixing with itself: max[*f*_*0,dg*_^*2*^*(d*_*ij*_*)*]=*f*_*0,dg*_^*2*^*(0)*=1. Here, *d*_*g*_ is the unit distance between two neighbouring sources on the same row or column and *d*_*ij*_ is the spatial distance between *v*_*i*_ and *v*_*j*_. Thus, the mixing function is identical for each point-source on the grid. Because the grid contains *n*^*2*^ sources (*n* rows×*n* columns), the mixing function *f*_*mix*_*(v*_*i*_*, v*_*j*_*) is a n*^*2*^×*n*^*2*^ matrix.

**Fig. 1:**
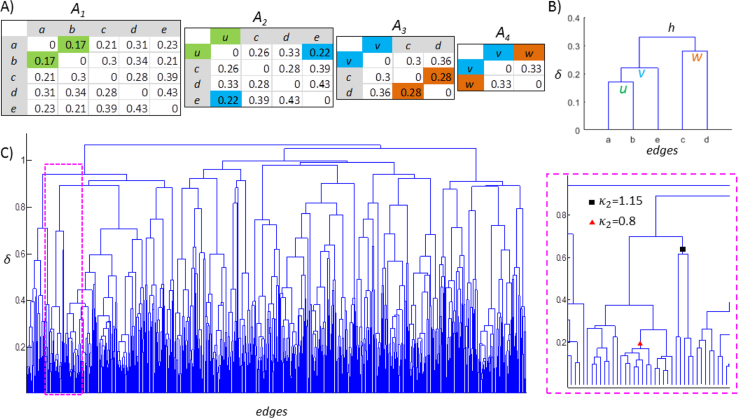
***A*** The similarity matrices produced during the iterative procedure of UPGMA. ***B*** The hierarchical tree represents the tied structure in *A*_*1*_. ***C*** Left: the tree of the random graph shown in Fig. 3B. Right: an inset of the tree. The inconsistency coefficient was computed with depth of 2.

#### 2.1.1 Simulation of a single interaction

To demonstrate how multiple SIs can arise from a single true interaction in reconstructed source space, we simulated one phase-lagged interaction (between *V*_*1*_ and *V*_*2*_, Fig. 1A) on a grid of 13×13=169 sources. On the grid, each source has unit distance from its immediate neighbours. The time-series of all sources, except for *V*_*2*_*,* consisted of 1000 samples of Morlet-filtered white noise (*f*_*c*_=10 Hz, Morlet *m*=5, sampling frequency *f*_*s*_=100 Hz). The time series of *V*_*2*_ was simulated to have a correlation coefficient of 0.9 with that of *V*_*1*_ with a 3 samples delay. The distance between *V*_*1*_ and *V*_*2*_ on the grid was set to 8.5*d*_*g*_, to ensure there would be no mixing between them. We then modeled linear signal mixing so that the signal measured at any vertex *v* was obtained as:

(S2) $$X_{v}^{\prime }=X_{v}\left(t\right)+\sum_{i=1}^{M}f_{mix}\left(v,v_{i}\right)X_{vi}\left(t\right)\;$$

where *X*_*v*_(*t*) is the original time series of a source *v,* the mixing function *f*_*mix*_ measures the signal mixing between two sources and is defined in Eq. (2) of [1], *X*_*vi*_(*t*) is the original time series of *v*'s neighbor *v*_*i*_, and *M* is the number of sources that have non-zero *f*_*mix*_(*v,v*_*i*_). We used these linear-mixing-contaminated time series to compute phase interactions with *iPLV* between all pairs of point-sources. For illustration, we retrieved the strongest 0.1% of *iPLV* edges Fig. 1B of [1].

#### 2.1.2 Multiple true interactions with various distances in mixing

To illustrate concurrent bundling of multiple hyperedges, we simulated six interacting sources (true edges) on a grid system of 20×20=400 sources (Fig. 1 of [1]). Using these six edges’ spatial adjacency on the grid, we paired them as “kin”, “nearby”, and “far”. Note that the “far” pair, edge *E*(*V*_*9*_*,V*_*10*_) and *E*(*V*_*11*_*,V*_*12*_), were not only far from each other but also far from all other edges. After applying the mixing function to the simulated time-series and computing the pairwise *iPLV*, we thresholded the resulting 400×400 source interaction matrix at 99.7th percentile, obtaining 239 significant edges that are visualized in Fig. 1D of [1].

### 2.2 Simulation on cortical source-space

We simulated the parcel time-series *X* of 1,000 randomly chosen ground-truth graphs on a 400-parcel cortical source-space obtained by iterative splitting [3] of the Destrieux atlas [4], [5], [6]. Virtual MEG measurements and source reconstruction were performed by forward- and inverse-modeling *X* into the reconstructed time series $\hat{X}$. The forward- and inverse-operators used here were derived from the visual working memory experiment described below. Finally, all-to-all FC of $\hat{X}$ was estimated with the imaginary part of the complex phase-locking value (*iPLV,* Wang et al., in press).

Each ground-truth graph contained 200 randomly connected nodes so that each node was connected only to a single other node. The narrow band time series *X(t, f*_*0*_*)*∈ℝ^m×n^ (*m=*1000 independent samples, *n=*400 parcels) of each ground-truth graph was simulated as follows:

1) 400 parcels were first randomly divided into two groups *V*_*1*_ and *V*_*2*_ as source and target nodes.
2) The nodes from *V*_*1*_ and *V*_*2*_ were next randomly paired to create 200 edges *E*, thus for each edge *e*_*k*_*=*{(*v*_*1*_^*(i)*^*,v*_*2*_^*(j)*^)∈*E| v*_*1*_^*(i)*^∈*V*_*1*_*; v*_*2*_^*(j)*^∈*V*_*2*_}, 1≤*k*≤200.
3) The time series of 200 source nodes in *V*_*1*_ were obtained by convoluting uncorrelated white noise time series with Morlet wavelets $w(t,f_{0})$, *f*_*0*_=10 Hz, Morlet *m*=5, *f*_*s*_=100 Hz.
4) For each edge *e*_*k*_(*v*_*1*_^*(i)*^*,v*_*2*_^*(j)*^), the time series of the target node *v*_*2*_^*(j)*^ was simulated to have a correlation coefficient of 0.9 with the time series of source node *v*_*1*_^*(j)*^ delayed by 3 samples.
5) *X(t, f*_*0*_*)* were finally decimated into 1000 independent samples before forward and inverse-modeling (see *Theory,* Wang et al., in press).

To identify significant *iPLV* edges in the ground-truth graphs, we also estimated null hypothesis graphs *G*_*rawH0*_ *by* simulating uncorrelated time series *X*_*H0*_∈ℝ^m×n^ (*m=*1000, *n=*400 parcels) and estimating their *iPLV* as done for the ground-truth graphs. A range of five thresholds was obtained from *G*_*rawH0*_ that corresponded to significance levels ranging from relaxed to strict, *i.e.*, *T*_*iPLV*_=*−log(p-value)*∈{*1.3, 2, 3, 4, 5*} (equivalent to *p*<from 0.05 to <0.00001).

### 2.3 The unweighted pair group method with arithmetic mean clustering

Here we illustrate the partitioning of the similarity matrix *S*_*E*_ with the unweighted pair group method with arithmetic mean (UPGMA). The UPGMA algorithm is an agglomerative hierarchical clustering method that builds a hierarchical tree through an iterative procedure to reflect the distance between pairs of objects in an adjacency matrix *A* [7]. In each iteration, two objects *p* and *q* with nearest distance *d*(*p*, *q*) were linked into a cluster. Here, *p* and *q* can be either an element from *A* or a cluster of elements from *A*, and the distance between *p* and *q* is defined as

(S3) $$d\left(p,q\right)=\frac{1}{n_{p}n_{q}}\sum_{x\in p}\sum_{y\in q}d\left(x,y\right)$$

where $n_{p}$ and $n_{q}$ are the number of elements in *p* and *q* respectively, *d*(*x, y*) is the distance between *x*, *y*, and *x*, *y* are elements from *A*.

Suppose that we have a matrix *A*_*1*_ derived from the edge-to-edge adjacency matrix (Fig. 1A), where each element is the distance between edges (*a*~*e*), and we intend to build a hierarchical tree to reflect the tied structure of similarity in signal mixing between all these edges.

First, we find the closest two edges in *A*_*1*_, *i.e., d(a, b)=0.17*, and combine them to form cluster *u.* Next*,* we compute the distance between *u* and the rest of edges *c*, *d* and *e* using Eq. (S3), and nearest objects in the updated matrix (*A*_*2*_) is *d*(*u, e*)=0.22. Hence we cluster *u* and *e* to *v.* By repeating this, eventually all the objects will be connected into a hierarchical tree (Fig. 1B), in which the height *δ* of a cluster on the tree is the distance between the two members of that cluster, *e.g.*, *δ*(*v*), the height of *v,* the distance of u and e, *is 0.22.*

The information about tree structure is used to partition the data into well separated clusters. If a cluster's height is close to its members’ height, then the members of the joined cluster at this level of hierarchy are very similar or “inseparable”. Otherwise, the members should be separated into two distinct clusters. This can be quantified using the inconsistency coefficient

(S4) $$\kappa_{\theta }=(\delta \;-\mu_{\delta })/\sigma_{\delta }$$

where *δ* is the height of a cluster, $\mu_{\delta }$ and $\sigma_{\delta }$ are the mean and standard deviation of the clusters height included at a given search depth *θ* below (and including) this cluster. For example, in cluster *h* (Fig. 1B), with *θ* of 2, ***μ***_*d=*_(*δ*(*h*)+*δ*(*v*)+*δ*(*w*))/3=0.277 and with *θ* of 3, ***μ***_*d=*_(*δ*(*h*)+*δ*(*v*)+*δ*(*u*)+*δ*(*w*))/4=0.25. The $\kappa_{\theta }$ of bottom tie clusters is zero.

After the tree is built, the inconsistency coefficient $\kappa_{\theta }$ can be computed for all clusters, and the cutoff limit (CL) defines the criterion by which the tree is partitioned into clusters, *e.g.*, CL=0.15 means those clusters whose $\kappa_{\theta }$ exceed the 85^th^ percentile will be partitioned into independent clusters.

In the example of the random graph in Fig. 6C, when performing bundling with a CL of 0.15 and a depth *θ* of 2, those clusters whose $\kappa_{2}$ is greater than 1.04 will be partitioned, *e.g.*, the cluster indicated by the black box (right panel, Fig. 1C). On the other hand, those clusters (and all of its branches) whose $\kappa_{2}$ is less than 1.04 will remain as a distinct cluster, *e.g.*, the cluster indicated by the red triangle. For more details about the UPGMA algorithm, please visit Matlab® Statistics and Machine Learning Toolbox™ (http://se.mathworks.com/discovery/cluster-analysis.html).

### 2.4 Workflow for testing the stability of hyperedge

**Fig. 2:**
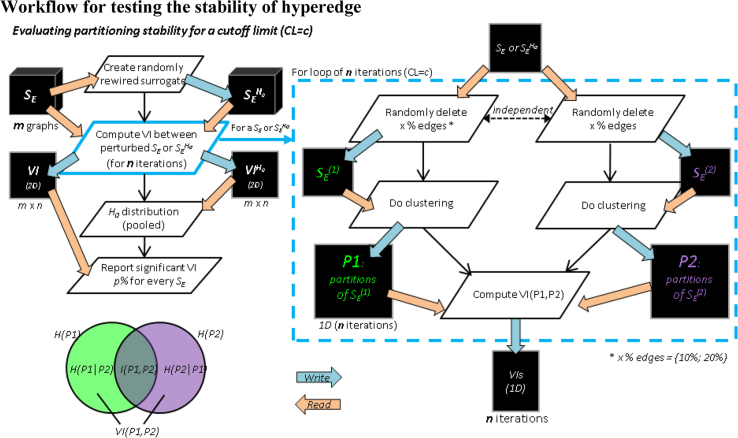
***Left***: Evaluating the stability of hyperedge clustering solutions. ***Left bottom***: a schematic illustrating the relation between entropies (H), mutual information (I) and variation of information (VI) between two partitioning solutions P1 and P2 (Meila, 2007). ***Right***: computing the VI between two perturbed version of a similarity matrix *S*_*E*_ (or its surrogate *S*_*E*_^*H0*^). The surrogates *S*_*E*_^*H0*^ were obtained by randomly rewiring the original similarity matrix. The independent perturbations to a similarity matrix are achieved by randomly deleting a small subset, *e.g.*, 10–20%, of the elements in the similarity matrix.

To ensure that the hyperedges are not random outcomes of partitioning the edge similarity matrix, we tested, at any given resolution, if the differences between the partitioning solutions of n randomly perturbed versions of a edge similarity matrix is statistically smaller than their surrogate counterparts. The workflow is described below (Fig 2):

### 2.5 Using the software and sample data to replicate the main results

First, download the sample dataset and the software package from the public repository given above and unzip the package to local disk. The package includes following contents, and the Labview code runs on the MS Windows operating system and requires Labview 2015 or newer and Matlab R2013 or newer.

**Fig. 3:**
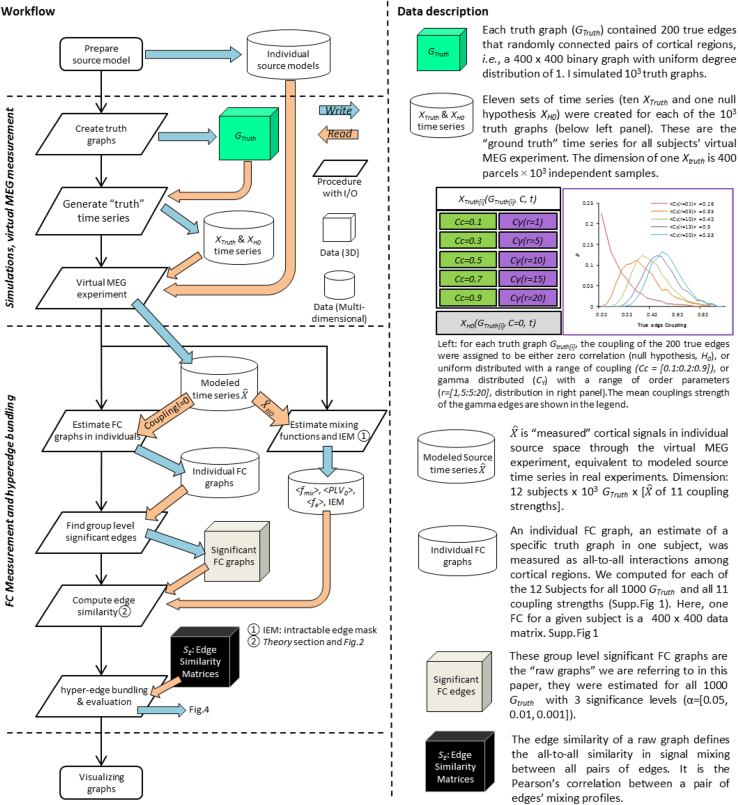
Workflow and data description of stimulation and hyperedge bundling on cortical source-space.
• The sample data: *Hyperedge_sample_data.zip* (for Labview GUI only), includes 10,000 truth graph templates, individual subject forward, inverse and fidelity optimized sparse collapsing operator [8]. These data were derived from the working memory experiment introduced below.
• Python source code: https://github.com/palvalab/hyperedges. This includes the hyperedge bundling core functions as well as a Python notebook demonstration. The software was written in Python from the Python Software Foundation (http://www.python.org/), version 2.7.

• The Labview GUI provides functions for “simulations, virtual MEG measurement” and “FC measurement and hyperedge bundling” (workflow, Fig. 3) to replicate the main results of hyperedge bundling paper. The GUI provides detailed instructions at each step. We offer both source code and complied executable files of the GUI. Source code: Hyperedge(LabviewSource).zip. Labview executable: Hyperedge_GUI_EXE.zip and Hyperedge_GUI_Setup.zip. Note: if you do not have Labview (or Labview runtime engine) installed on your PC, you will need to use the Setup program. Detailed instructions for simulations and tests can be found on the GUI.

### 2.6 Visual working memory (VWM) experiment

We illustrated the application of hyperedge bundling to real MEG data using *iPLV* raw graphs obtained from a VWM experiment. In the VWM task, subjects memorized one feature in the Sample stimulus and reported whether the memorized features were the same in the Test stimulus presented 2 seconds later. Data preprocessing and group statistics were carried out in the same manner as in our earlier studies and are described in these studies and their supplementary material [3], [9], [10]. The raw graphs contained the connections with significantly stronger phase correlations during the VWM retention period than during pre-stimulus baseline.

#### 2.6.1 Experimental paradigm

**Fig. 4:**
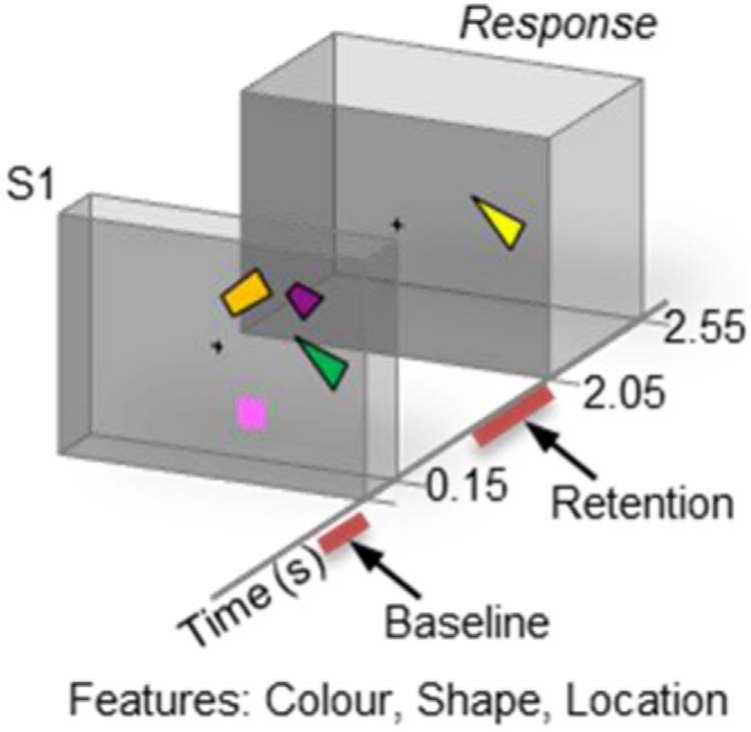
The working memory experimental paradigm.

To demonstrate the hyperedge bundling approach can be applied to real MEG/EEG data, we estimated raw FC graphs from a visual working memory (VWM) MEG experiment. The paradigm of the VWM experiment is descrbied below (Fig. 4):

In each trial, subjects were first shown a sample stimulus (S1) that contained 2 or 4 objects (Load2 and Load4) in either left or right visual field. The objects were 3- to 6-sided polygons with distinct colors. After a 2.05 s retention period (black screen with a center fixation), a test stimulus of one object (S2) was displayed in the same visual field where S1 was shown. Subjects made a forced-choice response to indicate whether the shape of S2 matched any one of the objects in S1. The response was a left or right thumb twitch, to indicate whether S2's shape matched any of S1's shapes. Object features were generated randomly so that each S1 and objects therein were unique, and the shape of S2 object always had a 50% probability of being identical with any of the object features in S1. The field of view was 7.3°×7.3° and the objects on average spanned an area of 0.65°×0.65°. The minimum center-to-center distance between the objects was 1.29° and maximum 3.87°. Prior to M/EEG recording, subjects were given clear instructions and performed at least one practice session. M/EEG recordings were carried out in 5 blocks. Each block comprised 160 trials. We estimated network phase synchrony for a baseline period (0.7–0.1 s prior to S1) and two retention periods (0.6–1.2 s and 1.2–1.8 s after S1) as indicated. For simplicity, we selected a subset of trials from the whole experiment where subjects made a correct response (indicating they remembered the shape the sample stimuli) in the Load4 condition.

#### 2.6.2 Subjects and recording

12 healthy subjects (29±6 years of age, mean±SD, 7 females) participated in the experiment. We acquired T1-weighted anatomical MRI scans for each subject at a resolution of ≤1×1×1 mm with a 1.5-T MRI scanner (Siemens, Germany). During the VWM experiment, concurrent M/EEG data were recorded with 204 planar gradiometers, 102 magnetometers, and 60 EEG electrodes (Elekta Neuromag Ltd, Finland) with a sampling rate of 600 Hz. The preprocessing of the M/EEG data was described in [10]. The projects were approved by the Ethical Committee of HUS and conducted in accordance with the Declaration of Helsinki.

#### 2.6.3 Cortical surface reconstruction and parcellation

We used FreeSurfer software (http://surfer.nmr.mgh.harvard.edu/) for volumetric segmentation of MRI images, reconstruction of the pial and the white and gray matter surfaces, and neuroanatomical labelling with the Destrieux atlas [4], [5], [11]. This atlas is comprised of 148 parcels covering the entire neocortex. To obtain a parcellation with a finer resolution and to decrease variability among parcel surface areas, those parcels that had largest mean area in the subject cohort were iteratively selected and split in two [3] until a total of 400 parcels was obtained [3], [12].

### 2.7 Forward operator, inverse operator and source reconstruction

We used MNE software (www.martinos.org/mne/) for MEG-MRI co-localization, creating individual source models with dipole orientation fixed to surface normal at 7-mm spacing, and preparing forward and inverse operators [13], [14], [15]. M/EEG sensor data were filtered using Morlet wavelets into 31 narrow-band frequency time-series covering frequency bands from 3 to 120 Hz with equal spacing on the log scale. Wavelet analysis was employed because it does not require the data to be stationary and therefore is more suitable for non-stationary neurophysiological signals than Fourier-based methods [16]. Inverse-operators were prepared for each frequency band with the noise covariance matrix χ estimated from pre-sample-stimuli baseline. For each subject, the sensor time series of these trials were projected into sources time series (6–8 k sources/subject) using Eq. (5) of [1] and *λ*^*2=*^0.05. These time series were collapsed into 400-parcel space using a sparsely-weighted-collapse-operator that was optimized to maximize source-reconstruction accuracy [8].

## 3 The mixing properties of the source space

**Fig. 5:**
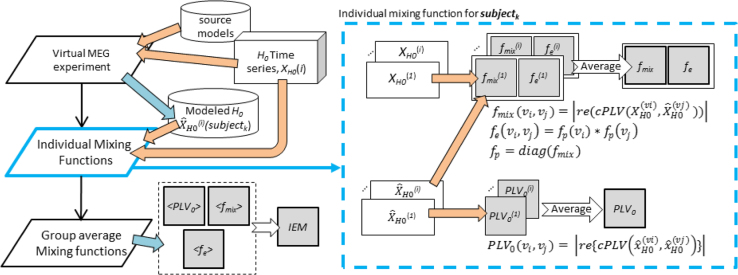
The workflow for computing the mixing properties. IEM: group-level intractable-edge-mask. *PLV*_*0*_: the residual spread function. *f*_*mix*_: the mixing function. *f*_*e*_: the edge fidelity function.

We first estimated the mixing properties (*i.e.*, *f*_*mix*_, *PLV*_*0*_, *f*_*p*_) for individual cortical source-space and next computed the group average (Fig. 5, left). The individual mixing properties were obtained by averaging the results over 10 iterations in each iteration, the virtual MEG measurement of one null hypothesis time series *X*_*H0*_^*(i)*^ would yield one set of raw mixing property estimates (in cyan box, Fig. 5).

**Fig. 6:**
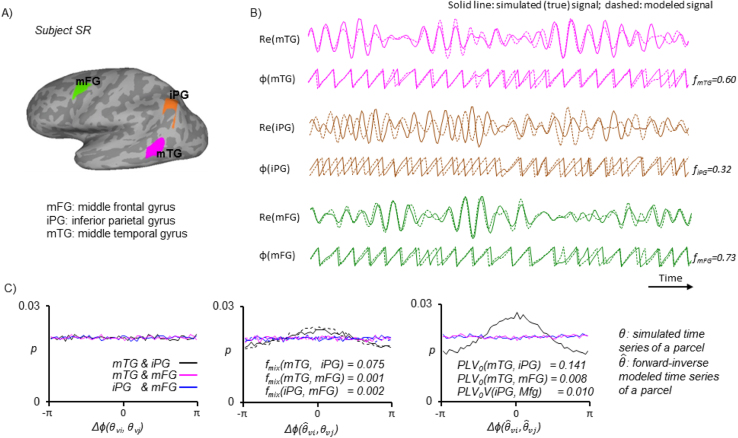
**A)** Three region-of-interests. ***B)*** The simulated and modeled signal amplitude (Re) and phase (ϕ) from these three regions. The fidelity estimates shown right to the phase time series indicate modeling accuracy. ***C)*** The distribution of phase difference between original and modeled null hypothesis time series.

We picked three regions of interest (ROIs) to illustrate the computation of mixing properties in one subject (Fig. 6A, B). Among these three ROIs, the medial frontal gyrus (mFG) is spatially distal from the other two ROIs, and therefore, the mixing between mFG and two posterior parcels can be ignored. The inferior parietal gyrus (iPG) and the medial temporal gyrus (mTG) are relatively close to each other, and therefore, there should be small but non-zero mixing between them. To demonstrate this, we aligned discrete phase time series of original and modeled null hypothesis time series from these parcel pairs, we subsequently subtracted sample-by-sample to obtain phase difference time series. The phase difference distributions were next computed for 50 bins between – π and π (Fig. 6).

First, phase differences between the original null-hypothesis time series of these three ROIs were uniformly distributed, and therefore *PLV* estimates were zero (Fig. 6C, left). However, there was a preferred phase difference at zero degree between original null hypothesis and modeled phase time series between ($\theta_{\mathrm{iPG}}$, ${\hat{\theta }}_{\mathrm{mTG}}$) as well as between ($\theta_{\mathrm{mTG}}$, ${\hat{\theta }}_{\mathrm{iPG}}$) indicating a bi-directional, non-zero mixing effect (*f*_*mix*_), between mTG and iPG (Fig. 6C, middle). Last, the phase difference between modeled time series of (${\hat{\theta }}_{\mathrm{mT}G}$, ${\hat{\theta }}_{\mathrm{iPG}}$) was even larger than that observed in Fig. 6C middle, indicating a large residual spread (*PLV*_*0*_) between them (Fig. 6C, right).

**Fig. 7:**
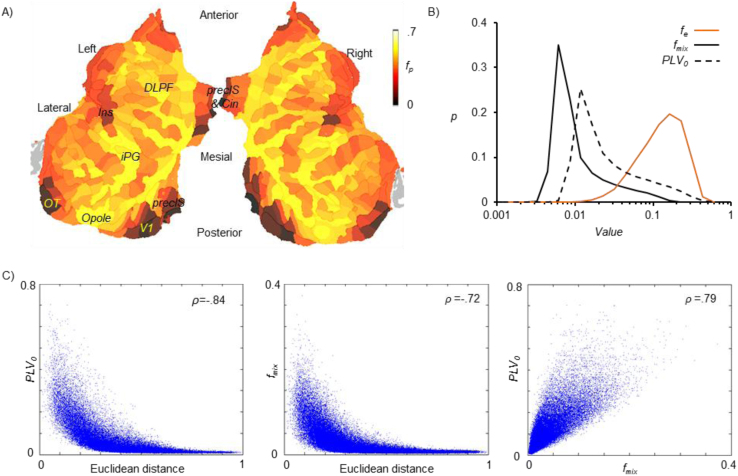
**A)** Group average parcel fidelity overlaid on a flattened 2D cortical map. ***B)*** Distribution of edge fidelity *f*_*e*_, mixing function, and residual spread *PLV*_*0*_ computed with 20 log-bins. ***C)*** Scatter plots showing correlation between parcel-to-parcel Euclidean distance, *PLV*_*0*_ and *f*_*mix*_(*v*_*i*_*,v*_*j*_). The Spearman's rank correlation coefficient (*ρ*) for each pair of metrics is shown on top right corner.

We observed that parcel fidelity *f*_*p*_ was spatially inhomogeneous across the cortical surface, and that low fidelity parcels were clustered in the medial and deep structures (Fig. 7A). The shape of the distributions of *f*_*mix*_ and *PLV*_*0*_ were similar (heavy-tailed) and *PLV*_*0*_ was greater than *f*_*mix*_ (Fig. 7B). Moreover, *f*_*mix*_ and *PLV*_*0*_ were both negatively correlated with parcel distance (Fig. 7C).

**Fig. 8:**
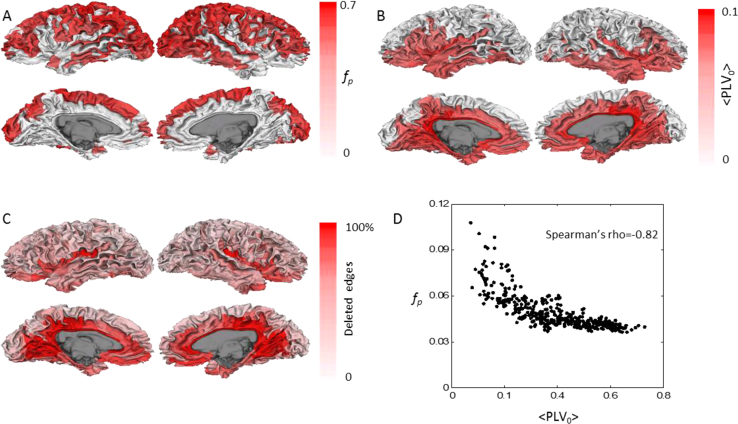
Group level mixing properties and the intractable-edge-mask ***A)*** Parcel fidelity, ***B)*** Parcel mean residual spread. Fig A and B were thresholded so that parcels with the strongest 50% value were shown. **C)** The percentage of deleted edges of each parcel defined in the intractable-edge-mask. For better visualization of sulci, we plotted data on a 3D white matter surface. ***D)*** Parcel fidelity as a function of residual spread.

We further inspected the spatial distribution of the group-level mixing properties. The high fidelity parcels were mostly situated on the dorsal and lateral sides of the brain and predominantly on gyri (Fig. 8A). In contrast, high residual spread parcels were located in deeper sources such as the cingulate, inferior occipital, temporal, fontal and insula (Fig. 8B). We next obtained the intractable-edge-mask (Methods of [1]) and deleted the 40% most poorly reconstructed interactions. Parcels with the most deleted edges are concentrated in the sulci and deep structures, including the cingulate, inferior occipital, temporal and insula. In addition, parcel fidelity and *PLV*_*0*_ were negatively correlated (Spearman's rank correlation coefficient *ρ*=−0.82, Fig. 8D), meaning that high fidelity parcels tend to have low residual spread.

## 4 The intractable edge mask (IEM)

**Fig. 9:**
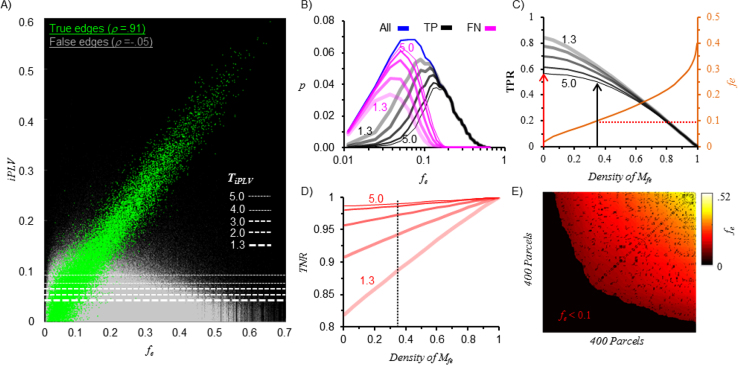
*Bias of modeling accuracy (i.e., edge fidelity) to iPLV estimate and the construction of the IME **A)** iPLV* estimates of true and false edges as a function of edge fidelity. Here, edges were pooled from 100 randomly chosen simulations (2×10^4^ true- and 8×10^6^ false-interactions); dashed lines indicate a range of threshold criteria ranging from 1.3 (relaxed) to 5 (strict). Above a given threshold green dots are true-positives (TP) and gray dots false-positives (FP), whereas below that threshold, green dots are false-negatives (FN) and gray dots true negatives (TN). ***B)*** Distribution of edge fidelity of true positive and false negative edges. ***C)*** The selection of the edge fidelity threshold for defining the edge fidelity mask *M*_*fe*_ (exclusion of edges estimated from poorly reconstructed loci). ***D)*** Tuning of the true negative rate. ***E)*** Visualization of the intractable-edge-mask in matrix form. The large dark area represents the deleted edges from poorly reconstructed loci (*f*_*e*_<0.1), the fragmented dark areas within the heat map indicates the “short-range” edges that were deleted due to high local mixing (quantified by null hypothesis *PLV*_*0*_).

We employed an IEM to delete edges connecting poorly reconstructed sources (Methods of [1]). Here we use an example to demonstrate applying IEM increases the quality of connectivity graphs. For example, in graphs of uniformly distributed edge coupling of 0.9, although all true interactions were simulated uniformly, the *iPLV* estimates of true positive edges were highly biased by edge fidelity *f*_*e*_ (Spearman's rank correlation coefficient *ρ=0.91,* Fig. 9A). This is because false negative (FN) edges (true edges that were rejected as non-significant) appeared to arise from loci of low edge fidelity (*f*_*e*_<0.2) (Fig. 9B).

If we define the edge fidelity mask *M*_*fe*_ using *f*_*e*_<0.1 (orange), 35% edges from the raw-graph are removed (Fig. 9C). This corresponds to a true-positive rate (TPR) of 0.5 in raw-graphs with *T*_*iPLV*_=5 (thinnest black curve), representing a small loss of TP compared to not applying *M*_*fe*_ (black vs red). The true-negative rate (TNR) of raw graphs with T_iPLV_=5 was 97% when applying the above mentioned *M*_*fe*_.

## 5 Mixing effects on measured individual FC graphs

After the virtual MEG experiment, the overall descriptive statistics of the FC graphs estimated in individual subjects were distorted due to mixing in the respective individual source spaces. For each truth graph, we simulated one set of null hypothesis time series *H*_*0*_ and ten sets of truth time series, which included two patterns of coupling strength (coupling of the true edges were uniform or gamma distributed with each pattern having 5 levels of strengths, see Fig. 3). Here, “o*ther*” edges refer to all *uncorrelated* signal pairs, and there are 8×10^4^ such “other than true” edges for a FC graph with dimension of 400×400.

**Fig. 10:**
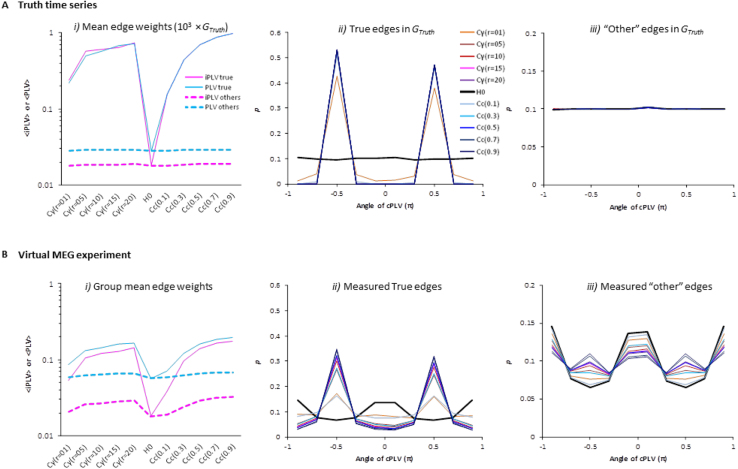
Demographics of the simulated graphs of truth time series (***A***) and after the virtual MEG experiment (***B***). Data pooled over 12 subjects, 100 randomly selected graphs per subject, ten levels of coupling strength and one *H*_*0*_ time series per graph.

In FC graphs estimated directly from the truth time series, the mean *iPLV* and *PLV* of true edges are much higher than other uncorrelated edges for all coupling patterns and strengths except for *H*_*0*_ time series where the true edges had uniform zero couplings (Fig. 10*A.i*). In these truth time series, <*iPLV*> and <*PLV*> are equivalent because coupling was simulated with a 90 degree phase-lag (Fig. 10*A.ii*) where *iPLV* is at its maximum and equal to *PLV*. On the other hand, mean *PLV* of *other* edges was slightly greater than *iPLV* because in uncoupled signal pairs, the mean phase difference is uniformly distributed (Fig. 10*A.iii*), and therefore, the projections of complex-valued *cPLV* to the imaginary axis are always smaller than the vector length of the *cPLV, i.e., PLV.*

The mean *iPLV* and *PLV* of true edges estimated after the virtual MEG experiment, *i.e.*, after linear mixing was introduced, were lower than those of the truth graphs yet higher than *other* uncorrelated edges (Fig. 10*A.i* vs. *B.i*). Moreover, the mean *PLV* of *other* edges after the virtual MEG experiment was nearly 3 times larger than in the truth graph (*i.e.*, an increase from 0.02 to 0.06) whereas there was a smaller increase in the mean *iPLV* of “*other*” edges.

In addition, we found a change in the phase-lag in the true edges after the virtual MEG experiment. All the true edges were simulated with π/2 phase-lag (Fig. 10*A.ii*), but we found a decrease in the number of true edges with π/2 phase-lag and an increase in the number of edges with in zero- or ±π phase-lag in all graphs (Fig. 10 *B.ii*). This change was found most prominently in graphs with weak coupling – as if the true interactions and mixing effects were two orthogonal forces competing to dominate the *cPLV* vector's angle (π/2 lag in true correlations vs. zero- and ±π phase-lag of mixing effect).

Finally, the phase-lag distribution of other uncorrelated edges in all 10 coupled graphs and in the *H*_*0*_ graphs deviated from the uniform distribution (Fig. 10*B.iii*). The most pronounced increase was observed in the zero- or ±π phase-lag edges due to mixing. This deviation from a uniform distribution explains 1) the systematical increase of *PLV* in all graphs that was seen earlier (implying the presence of a large amount of artificial interactions), and 2) the fact that the zero- and or ±π phase-lag (*i.e.*, artificial interaction) insensitive *iPLV* was less inflated than *PLV*. Moreover, there was also a slight increase in the number of edges having π/2 degree lag edges, which is a sign of spurious interactions that are “ghosting” the π/2 degree lag true interactions.

Note that, even in the most strongly coupled graphs (C_c_=0.9), the increase in the number of edges with π/2 degree lag was small (0.015 in both ±90). However, considering the large population of “other” edges (8×10^4^), a mere 0.03 increase in FPR means 2400 false positive spurious edges, overwhelming the true edges by a ratio of 12.

## Funding sources

This research was funded by the Academy of Finland grants 266745 and 281414. The funding bodies had no role in the design, data acquisition and analysis, decision to publish, or preparation of the manuscript.

## References

[1] Wang S., Lobier L., Siebenhühner F., Puoliväli T., Palva S., Palva J. (2018). NeuroImage.

[2] J.M. Palva, S.H. Wang, S. Palva, A. Zhigalov, S. Monto, M.J. Brookes, J.M. Schoffelen and K. Jerbi, NeuroImage 2018, 10.1016/j.neuroimage.2018.02.032. 29477441

[3] Palva J.M., Monto S., Kulashekhar S., Palva S. (2010). Proc. Natl. Acad. Sci. USA..

[4] Dale A.M., Fischl B., Sereno M.I. (1999). Neuroimage.

[5] Fischl B., Salat D.H., Busa E. (2002). Neuron.

[6] Destrieux C., Fischl B., Dale A., Halgren E. (2010). Neuroimage.

[7] Sokal R., Michener C. (1958). Univ. Kans. Sci. Bull..

[8] Korhonen O., Palva S., Palva J.M. (2014). J. Neurosci. Methods.

[9] Rouhinen S., Panula J., Palva J.M., Palva S. (2013). J. Neurosci..

[10] Honkanen R., Rouhinen S., Wang S.H., Palva J.M., Palva S. (2015). Cereb. Cortex.

[11] Fischl B., Sereno M.I., Dale A.M. (1999). Neuroimage.

[12] Palva S., Kulashekhar S., Hamalainen M., Palva J.M. (2011). J. Neurosci..

[13] Hamalainen M.S., Sarvas J. (1989). Biomed. Eng. IEEE Trans..

[14] Hamalainen M.S., Ilmoniemi R.J. (1994). Med. Biol. Eng. Comput..

[15] Lin F.H., Belliveau J.W., Dale A.M., Hamalainen M.S. (2006). Hum. Brain Mapp..

[16] D.B. Percival, A.T. Walden, Cambridge University Press, New York, NY, 2000.

